# Understanding smartphone use patterns in higher education: A latent class approach to behavioral and health risk typologies

**DOI:** 10.1371/journal.pone.0337918

**Published:** 2025-12-02

**Authors:** Ramón Ventura Roque Hernández, Rolando Salazar Hernandez, Adán López Mendoza

**Affiliations:** Faculty of Commerce, Administration, and Social Sciences, Autonomous University of Tamaulipas, Nuevo Laredo, Tamaulipas, México; Tarbiat Modares University Faculty of Medical Sciences, IRAN, ISLAMIC REPUBLIC OF

## Abstract

**Introduction:**

The widespread use of smartphones among university students has raised concern because of their potential effects and the need to detect profiles of problematic use. This study aimed to identify, characterize and differentiate different profiles of smartphone users in a sample of university students on the basis of variables such as use, nomophobia, risk and sociodemographic characteristics.

**Methods:**

A total of 681 university students participated. A total of 681 university students participated in this study. The sample was recruited using a non-probabilistic, convenience sampling method. Latent class analysis -LCA- was performed to identify profiles from variables that included smartphone use patterns such as daily hours, messaging, social networks, browsing, history of technology adoption, situational use, NMPQ nomophobia questionnaire -a scale designed to assess the fear of being without a smartphone-, and reported consequences such as accidents, visual or musculoskeletal problems. The resulting classes were compared in subsequent analyses using chi-square tests for categorical variables and Mann‒Whitney U tests for ordinal variables.

**Results:**

LCA revealed two clearly differentiated user profiles. Class 1 (n = 348) grouped users with moderate use and less exposure to risks and was characterized by shorter daily use of smartphones (mean = 5.46 hours), significantly lower scores on the total scale of nomophobia (mean NMPQ = 65.4 out of 140 possible points, moderate level), a lower frequency of accidents reported due to mobile use and lower reports of visual and musculoskeletal health problems. Class 2 (n = 333) grouped users with high digital involvement and multiple vulnerabilities and showed a significantly more intensive use pattern (mean = 11.01 hours per day), higher levels of nomophobia (mean NMPQ = 74.3 out of 140 possible points, moderate level), and a higher frequency of accidents and major visual and musculoskeletal health problems.

**Conclusion:**

While both groups of undergraduate students could benefit from awareness and training programs, interventions could be differentiated and designed to mitigate the risks associated with problematic smartphone use. These findings provide evidence for higher education institutions and health professionals in the development of programs aimed at promoting digital well-being among university students.

## Introduction

In the last two decades, the use of smartphones has become an increasingly ubiquitous practice in daily life [1], especially among young university students, who have personal, social, academic and work interactions with the support of their smartphones. Thus, these devices have radically transformed the modes of social interaction, business, access to information, learning and entertainment, configuring new environments that integrate the various dimensions of daily life in the same digital platform. The benefits of using smartphones have been evident in terms of connectivity, flexibility and access to knowledge. However, there has also been a growing increase in the risks associated with its excessive or dysfunctional use, especially in young populations [2]. In this way, digital well-being has emerged as an up-and-coming, necessary and increasingly interesting area of research that is composed of various interconnected aspects that require further study to promote the health of users of digital technologies [3].

Intensive and inappropriate use of smartphones has been associated with a series of adverse consequences [4]. These consequences are especially relevant for university students because of their high exposure to and dependence on devices for both academic and recreational activities. Within this context of problematic use in higher education lies nomophobia, which is defined as the fear or discomfort of not being able to use a cell phone [6]. It represents a growing concern in the digital age. Nomophobia is the term that brings together feelings of anxiety and its consequences in users because they are not close to their smartphones [7]. According to Luo et al. [6], nomophobia is a contemporary malaise that refers to the inappropriate use of smartphones, which can have serious consequences for the physical and mental health of university students.

Mudgal et al. [8] conducted a systematic review and meta-analysis on the prevalence and severity of nomophobia among medical students in India, analyzing 24 studies with 7,172 participants. The results revealed a pooled prevalence of mild nomophobia of 25%, moderate nomophobia of 59%, and severe nomophobia of 14%. The mean nomophobia score was 77.48, with significant variations across different states of India.

In Portugal, a study with 194 young people between the ages of 18 and 30 years revealed that 100% of the participants had some type of nomophobic symptomatology, with 59.3% showing moderate nomophobia and 24.2% showing severe nomophobia [9]. These findings are consistent with another Portuguese study that reported that 100% of the subjects had nomophobia, with 62% at a moderate level and 22% at a high level [10].

However, smartphone use and nomophobia are not homogeneous phenomena but are modulated by various individual and behavioral characteristics. For example, gender differences in smartphone use and nomophobia have been consistently documented in the literature. Dissing et al. [11] conducted a longitudinal study with 816 young adults and analyzed more than 795,000 interactions with smartphones. They reported that while smartphone interactions were associated with lower levels of loneliness and fewer depressive symptoms overall, there were significant differences between genders. In men, a high level of smartphone interaction was associated with fewer sleep disturbances. In contrast, in women, a high level of interaction was associated with increased loneliness and depressive symptoms over time. That is, the mental well-being of women tends to have more negative effects with the intensive use of smartphones.

Pinheiro and Maia [9] reported that women had significantly greater levels of nomophobia (mean NMPQ = 109.35) than men did (mean NMPQ = 71.66). Similarly, Maia and Sousa [10] reported that women presented higher levels of both nomophobia (mean NMPQ = 176.28) and phubbing (mean = 167.22) than men did (mean NMPQ = 124.73 and mean = 141.93, respectively).

In a study with nursing students, Bernabé-Mateo et al. [12] the mean nomophobia score was higher in women (NMPQ 70.33 ± 25.82) than in men (NMPQ 65.76 ± 27.06). Furthermore, in men, alcohol consumption was positively correlated with levels of nomophobia.

During the COVID-19 pandemic, Saadeh et al. [13] reported that women had significantly higher smartphone addiction scores than men did and that being a woman was identified as a factor associated with greater smartphone addiction. Additionally, recently, Alodhialah et al. [14] identified female gender as a significant predictor of nomophobia.

The intensity of smartphone use also varies considerably across different populations and contexts. For example, Saadeh et al. [13] reported that 85% of college students increased their smartphone use during the COVID-19 quarantine, with approximately 42% using their smartphones for more than 6 hours a day. Notably, three-quarters of the students expressed their desire to reduce their use of smartphones.

Ali and Matarneh [15] conducted research with 636 smartphone users at various universities in Jordan. They reported that the participants used their device an average of 7.9 hours per day, with a total range of one to 24 hours of daily use. Alodhialah et al. [14] reported that 42.9% of college students had owned a smartphone for 4–6 years, and 24.3% had used it for more than 6 years. In terms of the number of devices, 62.9% owned one smartphone, 27.1% owned two, and 10% owned more than two smartphones.

Given this behavioral and demographic complexity, the literature has identified a wide range of consequences and correlates that must be considered for a comprehensive understanding of the phenomenon.

Ahn et al. [16] identified significant positive associations between problematic smartphone use and depression, social interaction anxiety, and sleep disorders. They also reported a significant negative association between problematic smartphone use and the amount of time users spend at home.

Pinheiro and Maia [9] reported positive and statistically significant correlations, with strong magnitudes, between nomophobia and anxiety, depression, and stress. They also reported a significant and negative correlation between nomophobia and age, indicating that younger subjects have higher levels of nomophobia.

Alodhialah et al. [14] identified female gender, the number of smartphones one owns, and years of smartphone ownership as predictors of nomophobia. The academic year showed a negative relationship, indicating that students in higher academic years experienced lower levels of nomophobia.

Saadeh et al. [13] identified factors associated with greater smartphone addiction, such as being female, studying scientific or medical careers, having a higher level of income, being in quarantine in an apartment without a garden, and living in urban areas.

Moreover, Sserunkuuma et al. [17] reported that the prevalence of being at risk of smartphone addiction was 45.72% in a sample of 269 medical students in Uganda. They also reported a positive and statistically significant relationship between the risk of smartphone addiction and depression.

Ali and Matarneh [15] reported that the gender of college students, the frequency of telephone calls, the number of calls made and received per day, and the number of text messages received per day were predictive factors of nomophobia. In this study, women experienced higher levels of nomophobia.

Additionally, phubbing is a relatively new term that unifies the words “phone” and “snubbing” in the English language. Phubbing refers to the behavior of concentrating on smartphones in face-to-face interactions with other people [26]. It is also understood as the act of snubbing or neglecting other people to pay attention to the smartphone. Phubbing has been studied in relation to nomophobia. For example, in the work of Sun and Yoon [26] with 266 university students, a positive relationship was found between smartphone dependence and fphubbing (friend phubbing), defined as phubbing when it occurs between friends. Maia and Sousa [10] reported positive and significant correlations between all the subscales of the nomophobia and phubbing instruments, and women presented higher levels of both nomophobia and phubbing.

Moreover, problematic smartphone use significantly affects academic performance. Alodhialah et al. [14] found a significant negative correlation between nomophobia scores and GPA, as well as between nomophobia and academic concentration. This suggests that students with higher levels of nomophobia tend to have lower academic performance and greater difficulty concentrating during academic activities. Similarly, another investigation by Rodríguez-García et al. [27] reported that students whose academic performance is affected by the use of mobile phones have higher rates of anxiety, nervousness, discomfort, fear or discomfort when not being able to use their smartphone.

The long-term use of smartphones significantly affects visual health as well. For example, Lopez-Choquegonza et al. [18] reported a high prevalence (78.1%) of computer vision syndrome (CVS) among health sciences students in Peru. The prevalence of CVS was greater among students with moderate and severe nomophobia. Participants with more frequent symptoms of CVS had higher scores on the NMPQ nomophobia questionnaire; for example, those who experienced double vision “often or always” had a mean NMPQ score of 78.8 ± 25.1, whereas those who never experienced double vision scored 60.7 ± 23.5. They also reported a higher prevalence of CVS in women (80.1%) than in men (75.1%). Similarly, they reported that those with eye pain had mean nomophobia scores of 76.9 ± 21.1, whereas those without eye pain had 59.1 ± 24.6. On the other hand, Leung et al. [19] closely investigated changes in corneal astigmatism and heterophoria after using smartphones while walking or sitting. They reported that using a smartphone while walking produced a change in H/V corneal astigmatism, a significantly greater effect than using a smartphone while sitting. They also concluded that the possible posterior optical effect and vergence after prolonged use of the smartphone should be considered.

Sleep disorders associated with the use of smartphones have been documented in several studies. Ahn et al. [16] found a significant positive relationship between problematic smartphone use and sleep disorders. The group with problematic use had significantly higher scores for sleep disorders than did the group without problematic use. According to a study conducted by Jahrami et al. [20] with 549 people, people’s dissatisfaction with their quality of sleep is closely related to both insomnia and nomophobia; in this way, this dissatisfaction could be used as an indicator to detect and diagnose nomophobia and insomnia. Bernabé-Mateo et al. [12] reported that women who were nomophobic or at risk of having it had poorer quality of sleep, whereas men with nomophobia had lower sleep efficiency. Poor quality of sleep increased the risk of nomophobia in their regression analysis. Furthermore, Dissing et al. [11] reported that a high level of interaction with smartphones was associated with fewer sleep disturbances in men but not in women.

Also, intensive use of smartphones has been associated with a notable decrease in the physical condition and activity of users. Ma et al. [21] investigated the association between the intensity of smartphone use and the physical performance of Chinese university students. They reported that the greater the number of hours of daily use of smartphones was, the lower the level of physical condition of the students. That is, students who used their smartphones more, took longer to complete walks of various lengths. This could be verified in both men and women. The average daily duration of smartphone use was 5.4 hours for men and 6.1 hours for women. Similarly, Le Steunf et al. [22] reported that, by reducing the time spent using smartphones, the average number of daily steps among participants increased. Therefore, they advised that the duration of smartphone use be reduced as a potential strategy for addressing physical inactivity and a sedentary lifestyle. In their research, the average duration of smartphone use was 3.08 hours per day.

Similarly, prolonged use of smartphones has been associated with musculoskeletal problems, particularly neck pain. Maayah et al. [23] conducted a cross-sectional study with 867 university students in Saudi Arabia to identify factors related to neck pain associated with the use of smartphones. The results revealed that the gender of the students, the time spent using their phones, the time spent on the devices to study and having a history of neck or shoulder pain were significant predictors of the duration of neck pain. These findings suggest that certain factors related to smartphone use may be associated with the severity and duration of neck and shoulder pain among college students. In their study, D’souza and Valechha [24] reported that cervical neuromotor control (cervical neuromotor control (CNC)) and subjective visual vertical control (subjective visual vertical (SVV)) were predominantly affected in a group of people aged 18–29 years who presented severe levels of nomophobia. This finding is important since the effects on CNC and SVV are associated with a lack of balance and an increased risk of falls and accidents in those who suffer them.

On the other hand, Useche et al. [25] conducted a study among 1,353 Mexican drivers; of them, 30% were 25 years old or younger. Almost all the participants (96.8%) recognized that the use of mobile phones while driving is a high-risk behavior. However, despite this, only 7.4% of them acknowledged completely avoiding its use while driving, and 22.4% said that they were very frequent users of the phone while driving. In this sense, a link was found between this frequency and the rates of collisions and near misses. Their findings indicate the need to further discourage the use of the phone while driving in a vehicle.

Despite extensive documentation of these factors separately, the prevailing analytical approach tends to examine variables in isolation, limiting the ability to identify patterns and risk profiles that integrate behavioral, psychological, and health dimensions simultaneously.

In this sense, a deeper understanding of the different modes of smartphone use is needed, especially through the identification of differentiated user profiles that integrate both behavioral patterns and physical, academic, and contextual aspects. Latent class analysis (LCA) is a statistical tool that is used to identify distinct subgroups within populations. However, few studies have applied this technique to typify the use of smartphones in university students, linking these profiles with health, academic, sociodemographic and behavioral variables. This gap in the literature highlights the need for a study that not only identifies these latent profiles but also has relevant practical implications for the design of preventive interventions, policies, and educational strategies aimed at promoting healthy technology use.

In this work, we included situational use patterns to reflect the context-dependent nature of smartphone behavior, nomophobia to capture the psychological dimension of problematic use, and self-reported physical and functional consequences (e.g., accidents, visual problems) to represent the real-world impact of excessive use. These variables were specifically chosen as the essential components for creating distinct and clinically relevant profiles that could not be identified using a single measure. By integrating these diverse dimensions, our study provides a more detailed and in-depth understanding of smartphone use and its associated risks in a university student population. This study aimed to address two research questions: 1. What are the latent profiles of smartphone users among university students based on their behavioral and contextual variables? 2. How do these emerging profiles differ in terms of physical health, academic performance, and sociodemographic variables?

In the following sections, the method followed for this research, and the results and their discussion are presented.

## Materials and methods

### Study design

A quantitative, cross-sectional and nonexperimental design with an exploratory person-centered approach [5] was used for this study to identify latent profiles of smartphone use among university students and to analyze the differences between classes in terms of sociodemographic, academic, physical and behavioral variables. LCA was used as a central analytical strategy because of its ability to model hidden typologies from multivariate response patterns.

### Participants

The sample consisted of 681 undergraduate students from a Mexican public university. The participants were selected through nonprobability convenience sampling. The inclusion criteria were as follows: (1) be officially enrolled in one of the three undergraduate academic programs of the Bachelor of Administration, Foreign Trade or Public Accounting, (2) be an active smartphone user, and (3) provide informed consent to participate in this study. The sample was diverse in terms of gender (57.3% women), age (mean = 21.09, standard deviation = 2.78), university degree and academic semester (semesters 1–12). Tables 1 and 2 provide useful information for the characterization of the participants.

**Table 1:** Distribution of Participants by Educational Program and Gender.

	Educational program			
Genre	Administration	Foreign trade	Public Accounting	Total
Female	137	162	91	390
Male	76	147	68	291
Total	213	309	159	681

**Table 2:** Characterization of the Age of the Participating Students.

Educational program	Genre	N	Missing	Mean	Standard deviation
**Administration**	**Female**	137	0	21.131	3.024
	**Male**	75	1	22.040	3.095
**Foreign trade**	**Female**	160	2	20.775	2.477
	**Male**	147	0	20.952	2.035
**Public Accounting**	**Female**	91	0	21.231	3.569
	**Male**	68	0	20.750	2.605

**Note:** Missing age values represent participants who did not disclose their age.

### Instrument

The instrument was a self-report questionnaire designed to capture a holistic perspective on smartphone use by integrating five key dimensions: sociodemographic and academic background, specific smartphone use behaviors, situational use patterns, levels of nomophobia, and self-reported physical and psychological risk indicators. Each of these sections was based on existing literature and was included to provide a better understanding of smartphone use that goes beyond simple measures of screen time and frequency.

A structured, self-administered and digitized questionnaire designed to collect information on the following dimensions was used:

1) Sociodemographic and academic variables: Age, gender, semester attended, academic program, and practice sports.
2) Smartphone use behaviors: years of use, number of cell phones owned thus far, average hours of daily use of the smartphone, in total, in messaging, social networks and browsing.
3) Situational use of the cell phone: Walking, during a face‒to‒face conversation, in a vehicle as a driver and as a passenger, eating, in the bathroom, during the night after going to bed. Response scale: Never, Sometimes, Always.
4) Levels of nomophobia: The Spanish version of the NoMoPhobia Questionnaire (NMPQ) was used [28], which was validated among the Spanish-speaking university population both previously [29,30] and in this investigation. The questionnaire consists of four factors, with twenty questions in total answered with a 7-point Likert response scale. The minimum score that can be obtained is 20; the maximum score is 140. To interpret these scores, the authors of the original instrument established the following levels: absence of nomophobia (20 points), slight nomophobia (21–59 points), moderate nomophobia (60–99 points), and severe nomophobia (100 points). points or more).
5) Risk indicators: self-reported accidents caused by distractions when one uses a smartphone, associated visual problems, musculoskeletal problems, academic problems, and use not allowed in class. Response scale: Yes/No.

The NMPQ questionnaire was subjected to a confirmatory factor analysis to determine if its original factorial structure could be preserved with the data sample collected for this research. The four original factors could be maintained since the factor loading of each item was greater than or equal to 0.79 in all the cases. The adjustment measures obtained are shown in Table 3. The high values of the Comparative Fit Index (CFI = 0.953) and the Tucker-Lewis Index (TLI = 0.945) indicate a good fit to the data, since both are well above the recommended threshold of 0.90 and very close to 0.95. On the other hand, the low value of the Standardized Root Mean Square Residual (SRMR = 0.038) and the Root Mean Square Error of Approximation (RMSEA = 0.081) further support the model’s good fit, as these values are below the conventional cutoff of 0.08. Finally, the Akaike Information Criterion (AIC) and Bayesian Information Criterion (BIC) are provided for comparison with alternative models, should they be tested.

**Table 3:** Adjustment Measures of the Confirmatory Factor Analysis for the NMPQ Questionnaire.

				RMSEA 90% CI			
CFI	TLI	SRMR	RMSEA	Lower	Upper	AIC	BIC
0.953	0.945	0.038	0.081	0.076	0.086	37602.430	37900.985

**Note:** CFI = Comparative Fit Index; TLI = Tucker-Lewis Index; SRMR = Standardized Root Mean Square Residual; RMSEA = Root Mean Square Error of Approximation; AIC = Akaike Information Criterion; BIC = Bayesian Information Criterion.

With respect to internal consistency, the McDonald ω indicator had a value of 0.97 when all the questions of the NMPQ questionnaire were included.

### Data collection

The data for this study were collected during the first semester of 2024 (March 1 to May 31) through a structured, multi-step process: 1) Recruitment and Collaboration: Professors from the academic programs were contacted to facilitate data collection. These professors, all of whom held a master’s degree and had at least five years of experience, were instrumental in distributing the survey link. 2) Informed Consent: Prior to beginning the survey, all participants were presented with a digital informed consent form. Their participation was contingent upon providing consent. 3) Data Collection: Participants accessed the survey via a unique hyperlink and its corresponding QR code. The electronic questionnaire was administered using Microsoft Forms, which ensured the anonymity and confidentiality of all responses. 4) Data Preparation: After collection, the raw data were coded and prepared for analysis. The dataset was formatted and imported into Jamovi statistical software for subsequent analysis.

### Statistical analysis techniques

The analyses were carried out in two stages using the Jamovi software (version 2.6.26) in its distribution for Windows.

#### Stage 1. Latent class modeling.

SnowLatent LCA for the Jamovi module (version 2.5.7) was used to run the LCA. Models with 2 to 4 classes were evaluated, and the optimal model was selected on the basis of the information criteria of Akaike information criterion (AIC), Bayesian information criterion (BIC), entropy, and interpretability of the classes. The two-class model presented the best balance between statistical fit and parsimony (AIC = 41343.69; BIC = 42795.756; entropy = 0.844) and was the closest conceptually to the intentions of this research.

#### Stage 2. Characterization and comparison between classes.

Each participant was assigned a class on the basis of the maximum probability of membership. Tests of differences between both classes were subsequently performed using frequencies and χ² tests for categorical variables, as well as scores and Mann–Whitney U tests for ordinal variables, which were not normally distributed. For the calculation of effect sizes, Cramer’s V was used in the case of the χ² tests and biserial correlation of ranks for the U tests. For categorical variables, standardized residuals were calculated to identify cells with significant differences between the expected and observed values. In all cases, a level of statistical significance of α = 0.05 was adopted.

### Ethical considerations

This specific project was reviewed and approved in October 2023, before the study began, by the Institutional Review Board of the Faculty of Commerce, Administration, and Social Sciences in Nuevo Laredo, Tamaulipas, and by the Institutional Review Board of Secretariat of Research and Graduate Studies at the Autonomous University of Tamaulipas in Ciudad Victoria, Tamaulipas (UAT/SIP/PIRP/2023/049). No other approval was sought since it was not necessary according to the institutional ethics standards of Autonomous University of Tamaulipas. Participants provided their electronic informed consent according to the institutional standards. The ethical principles of the Declaration of Helsinki were respected, guaranteeing confidentiality, anonymity, voluntary participation and the right to withdraw consent at any time. No sensitive data were collected.

## Results

### Identification of latent classes

Through LCA, two differentiated profiles of 681 university students using smartphones were identified. The two-class model showed an adequate fit to the data (*log likelihood* = −20,350.84; AIC = 41,343.69; BIC = 42,795.75), with a high level of entropy (0.844), which indicates good separation between the classes. With respect to the marginal prevalence, Class 1 represented 50.6% of the sample, and Class 2 represented 49.4%.

### Characterization and comparison of latent classes

Class 2 participants reported considerably more intensive smartphone use than Class 1 participants did, both in terms of daily duration and specific activities. For this reason, Class 1 was named “People with moderate and functional use of the smartphone”, and Class 2 was named “People with intensive and problematic use of the smartphone”. Tables 4 and 5 present the statistical comparisons of the ordinal and nominal variables between the two latent classes, respectively. Both tables include p-values and measures of effect size to assess the statistical significance and magnitude of the differences. A p-value less than.05 was considered statistically significant. Additionally, larger effect sizes indicate stronger associations or larger differences between the groups. Finally, a comprehensive summary comparing both latent classes is provided in Table 6.

**Table 4:** Comparison of Ordinal Variables between Latent Classes with the Mann‒Whitney U Test.

Variable	U	P value	Effect size (r)
Hours of daily use	13724	<.001	0.762
Hours of messaging	15005	<.001	0.741
Hours on social media	14010.50	<.001	0.758
Hours in navigation	21299	<.001	0.632
Number of cell phones	42291	<.001	0.270
Age	51682.5	0.022	0.100
Academic semester	51046.5	0.007	0.119
Year they started using their smartphone	51112.5	0.007	0.118

**Table 5:** Comparison of Nominal Variables between Latent Classes with the χ² Test.

Variable	χ²	gl	P value	Effect size (Cramer’s V)
Genre	8.03	1	0.005	0.109
Educational program	7.674	2	0.022	0.106
Nomophobia level according to NMPQ	16.60	3	<.001	0.156
Musculoskeletal problems	22.00	1	<.001	0.180
Visual health problems	24.89	1	<.001	0.191
You have had a careless accident when using your smartphone	43.16	1	<.001	0.252
Using your smartphone after going to bed	37.72	2	<.001	0.235
Use while walking	87.10	2	<.001	0.358
Use during face-to-face conversation	49.28	2	<.001	0.269
Use while driving	26.26	2	<.001	0.196
Use as a passenger (nondriver)	37.76	2	<.001	0.235
Use while eating	40.91	2	<.001	0.245
Use in class sessions	7.934	1	0.005	0.108
Use in the bathroom	25.51	2	<.001	0.194

**Table 6:** Comparative Summary of Characteristics with Statistically Significant Differences between the Two Classes Identified.

Dimension	Class 1 People with moderate and functional use of the smartphone	Class 2 People with intensive and problematic use of the smartphone
Prevalence	50.6%	49.4%
Overrepresented gender (Observed frequencies> Expected frequencies)	Male	Female
Overrepresented educational program (Observed frequencies > Expected frequencies)	Public Accounting	Foreign trade
Average age	20.96 years (SD = 2.9)	21.21 years (SD = 2.6)
Average academic semester	4.89 (SD = 6)	4.18 (SD = 12.9)
Daily use	Average: 5.46 h (SD = 2.1)	Average: 11.02 h (SD = 4.6)
Use of messaging	Average: 2.63 h (SD = 2.2)	Average: 8.24 h (SD = 5.39)
Use of social networks	Average: 2.66 h (SD = 1.6)	Average: 7.96 h (SD = 5.1)
Use for web browsing	Average: 1.83 h (SD = 1.7)	Average: 6.12 h (SD = 5.5)
Number of cell phones	Average: 4.41 (SD = 1.8)	Average: 5.26 (SD = 1.9)
Starting year using smartphone	2013.39 (SD = 2.34)	2012.95 (SD = 2.4)
Musculoskeletal problems	18.7% of the class	34.5% of the class
Visual health problems	26.8% of the class	45% of the class
They have suffered careless accidents associated with the use of the smartphone	5.7% of the class	23.4% of the class
Average level of nomophobia (According to the NMPQ questionnaire)	Moderate-Low (Average = 65.43)	Moderate-Intermediate (Average = 74.32)
Night use after bedtime	28% “Always” 57.8% “Sometimes” 14.1% “Never”	51% “ Always “ 40.5% “Sometimes” 8.4% “Never”
Use while walking	0% “Always” 74.4% “Sometimes” 25.6% “Never”	15% “ Always “ 77.8% “Sometimes” 7.2% “Never”
Use during face-to-face conversation	0.3% “ Always “ 50% “Sometimes” 49.7% “Never”	7.8% “ Always “ 63.7% “Sometimes” 28.5% “Never
Use while driving a vehicle	0.9% “ Always “ 16.1% “Sometimes” 83% “Never”	4.5% “ Always “ 28.5% “Sometimes” 67% “Never”
Use as a passenger in a vehicle	21.6% “Always” 70.1% “Sometimes” 8.3% “Never”	43.5% “Always” 51.1% “Sometimes” 5.4% “Never”
Use while eating	16.7% “ Always “ 61.2% “Sometimes” 22.1% “Never”	35.7% “ Always “ 54.4% “Sometimes” 9.9% “Never”
Use in the bathroom	14.4% “Always” 47.4% “Sometimes” 38.2% “Never”	29.1% “Always” 45% “Sometimes” 25.8% “Never”
Use during class sessions	Less frequent use (35% do use it during class)	Greater frequency of use (46% do use it during class)

### Academic and demographic variables

Relevant sociodemographic differences were observed. With respect to gender, a greater percentage of female participants were found than expected in Class 2 (62.7%) vs. 52% in Class 1, *χ²* = 8.03, *p* = .005. Class 2 members were slightly older than those in Class 1 (mean = 21.21 years vs. mean = 20.96 years in Class 1), *p* = .022, r = 0.10. With respect to the academic semester, the students in Class 1 tended to take more advanced semesters (average = 4.89 vs. average = 4.18 in Class 2), *p* = .007, r = 0.11. With respect to the academic program, Public Accounting students were more associated with Class 1, whereas Foreign Trade students were concentrated in Class 2 (*χ²* = 7.67, *p* = .022). On the other hand, no significant differences were found in the practice of physical activity (*χ²* = 0.707, p = 0.40) or in the presence of self-reported academic problems (*χ²* = 0.204, p = 0.65) between the two classes.

### Smartphone usage patterns

The following differences in use patterns were statistically significant and had large effect sizes: Hours of daily use: Class 2 = 11.02 h, Class 1 = 5.46 h, *U* = 13724, *p* < .001, *r* = 0.762; Instant messaging: Class 2 = 8.24 h, Class 1 = 2.63 h, *U* = 15005, *p* < .001, *r* = 0.741; Social networks: Class 2 = 7.96 h, Class 1 = 2.66 h, *U* = 14010, *p* < .001, *r* = 0.758; Web browsing: Class 2 = 6.12 h, Class 1 = 1.83 h, *U* = 21299, *p* < .001, *r* = 0.632. Additionally, the students in Class 2 reported having a greater number of mobile phones throughout their lives (mean = 5.26 vs. 4.41; *p* < .001, r = 0.27) and began to use them on average slightly earlier (mean = 2012.9) than did the students in Class 1 (mean = 2013.38) (*p* = .007, r = 0.11).

### Physical and functional impairments

In terms of physical consequences, Class 2 students presented significantly greater levels of musculoskeletal discomfort (*χ²* = 22.00, *p* < .001, V = 0.18), impaired visual health (*χ²* = 24.89, *p* < .001, V = 0.19) and accidents related to distractions from smartphone use (*χ²* = 43.16, *p* < .001, V = 0.25). On the other hand, significant differences were also observed in the levels of nomophobia according to the total score of the NMPQ questionnaire. Class 2 presented more cases of severe nomophobia than expected (*χ²* = 16.60, *p* < .001, V = 0.15), whereas class 1 presented fewer severe cases than expected.

### Risk behaviors associated with the use of smartphones

Students in Class 2 reported a higher frequency of smartphone use in the following risky or dysfunctional contexts: nighttime use after going to bed: 51% always did so in Class 2 vs. 28% in Class 1 (*χ²* = 37.72, *p* < .001, V = 0.235); use when walking: 15% in Class 2 vs. 0% in Class 1 (*χ²* = 87.10, *p* < .001, V = 0.358); use during face‒to-face conversations: 7.8% in Class 2 vs. 0.3% in Class 1 (*χ²* = 49.28, *p* < .001, V = 0.269); use when driving vehicles: 4.5% in Class 2 always do it vs. 0.9% in Class 1, 28% in Class 2 sometimes do it vs. 16% in Class 1 (*χ²* = 26.26, *p* < .001, V = 0.196); use when being a passenger in a vehicle: they always do 43.5% in Class 2 vs. 21.5% in Class 1 (*χ²* = 37.76, *p* < .001, V = 0.235); use while eating: in Class 2, 35.7% always use it, while in Class 1 only 16.7% (*χ²* = 40.91, p < .001, V = 0.245); use in the bathroom: in Class 2, 29.1% always use it, while in Class 1, only 14.4% (*χ²* = 25.510, p < .001, V = 0.19); use in classes: in class 2, 46% do use it in classes, while in class 1 only 35% (*χ²* = 7.93, p = 0.005, V = 0.108).

## Discussion

The main finding of this study is the identification of two distinct latent profiles of smartphone use among university students: Moderate and Functional Users (Class 1) and Intensive and Problematic Users (Class 2). This finding directly addresses the study’s primary aim of identifying and characterizing user typologies and reveals that the student population is not homogeneous in its digital behavior. The two classes differed significantly across behavioral, sociodemographic, academic, and health-related variables, confirming that these profiles represent meaningful distinctions in smartphone use and its associated consequences.

### Interpretation of latent profiles

The profiles reveal a strong dichotomy in smartphone-human interaction. *Intensive and problematic users* (Class 2) are characterized by markedly longer use of the smartphone, often exceeding 11 hours, along with high engagement in messaging, social networks and internet browsing. Conversely, *Moderate and Functional Users* (Class 1) exhibit a significantly more measured usage pattern, averaging approximately 5.5 hours per day, reflecting a more balanced time distribution and lower overall exposure to the device. This stark contrast in duration and frequency forms the behavioral basis for the differentiated risk assessment provided by the LCA.

The intensive use of Class 2 translates directly into a set of adverse physical and psychological outcomes. Notably, these users reported significantly higher levels of musculoskeletal and visual problems. They also exhibited a significantly greater frequency of accidents related to distraction caused by the use of the device, confirming that their use is not only high but objectively maladaptive and risky. Furthermore, Class 2 reported elevated levels of nomophobia, including a greater number of severe cases. These findings strongly reinforce the existing evidence that intensive smartphone use is intrinsically linked to objective risks to the health and physical well-being of users [4].

From a self-regulation perspective, the students in Class 2 are defined by the pervasive and dysfunctional context of their smartphone use. They showed a greater frequency of smartphone use in high-risk and socially inappropriate contexts, such as during face‒to-face conversations, while walking, driving or as passengers, when eating, while going to the bathroom, and after having gone to bed for the night. This pervasive pattern reflects a possible loss of self-regulation that is congruent with high levels of nomophobia. Previous studies have identified this persistent pattern of digital dependence as a factor that severely deteriorates the quality of sleep, increases stress, and causes significant social interference [12,16], underscoring the necessity of integrated intervention approaches targeting both the time spent and the where and when of device engagement.

### Demographic and academic differences

At the sociodemographic level, the overrepresentation of women in Class 2 is a relevant finding. This result is consistent with literature suggesting a greater tendency toward emotional and communicational use of smartphones in women [9,10,12–14], which may increase the vulnerability to anxiety over disconnection that defines this high-risk class. In academic terms, the finding that Foreign trade students were strongly associated with Class 2, while Public Accounting students associated more with Class 1, suggests that the cultural dynamics or specific communication demands of certain curricula might predispose students to higher dependency patterns.

Furthermore, the finding that intensive users tended to be in slightly earlier semesters suggests that the initial stages of the university training cycle represent a period of elevated vulnerability. This is consistent with previous findings, where the most intensive users are younger [9] or are in the first semesters of their professional studies [14]. This suggests that the adaptation to new academic loads and the need for self-management in the university context may be a critical period where problematic usage patterns are established, highlighting a key window for preventive interventions.

### Practical implications

The results of this study have relevant implications for public health, university policy and psychoeducational intervention. The identification of differentiated profiles of smartphone use allows progress toward the design of personalized intervention strategies, which consider the degree of risk associated with the patterns of use. Therefore, public screening tools are also advisable.

Digital education, technological hygiene and time management programs could be especially beneficial for students in Class 2, who have intensive use of smartphones and a greater risk associated with problem behaviors. For this group, interventions should be more direct, or even personalized, and include individualized counseling, digital boundary-setting techniques, the management of nomophobia, awareness of risks and accidents, and the promotion of disconnected activities that promote general well-being.

On the other hand, students in Class 1, characterized by more moderate use of smartphones, are not exempt from potential risk and represent an opportunity for direct and indirect prevention. For this group, strategies could focus on promoting healthy digital habits and early awareness of the risks of overuse. This could include, for example, general information campaigns on the impact of smartphone use on academic performance, sleep quality and social relationships. Campaigns could be held at specific events or during curricular or extracurricular activities.

Workshops could also be organized to raise awareness and learn about setting personal limits with technology, conscious use, and identifying the first signs of problematic use before they escalate to more severe levels. In the same way, extracurricular activities and study environments free from technological distractions could be promoted, integrating the promotion of digital disconnection as part of balanced and healthy university life. Universities could also consider and promote the monitoring of smartphone use as part of their student wellness strategies, paying special attention to the technological dimension in orientation and tutoring programs.

This dual approach, which addresses intervention in the highest risk groups and prevention in those with more moderate use, will allow university institutions to develop a comprehensive and scalable strategy to promote a healthier and more productive relationship with technology among their students.

On the other hand, any intervention strategy or preventive policy designed in the university environment must transcend the exclusive dependence of one-dimensional or average measurements, such as, for example, the total score of the NMPQ questionnaire. In the context of this work, Class 2, characterized by significantly intensive use and greater negative consequences for health and safety, presented an average NMPQ score at the moderate level of nomophobia, similar to that of Class 1. This is a limitation of the aggregate scales, which prevents characterization of the real complexity of the problematic use of the smartphone. For this reason, it is advisable to integrate multiple indicators — including target use patterns, behavioral risk variables, and one’s perceptions of health and well-being — for a more comprehensive evaluation. Additionally, it is important to perform a cautious interpretation of all self-report questionnaires, since social desirability or a lack of awareness of one’s own behavior may underestimate the real severity of the problem in populations with high levels of risk. Consequently, a multifactorial approach is recommended to design truly personalized and effective interventions.

## Contributions

This research contributes to the health areas of psychology, education and public health and establishes a typology of smartphone use on the basis of two clearly differentiated profiles in the university student community. The typology includes sociodemographic, academic, usage pattern and health variables. Thus, this study recognizes the use of smartphones as a multidimensional phenomenon that spans academic, social, physical and psychological dimensions. The findings demonstrate a direct relationship between the intensity of smartphone use and the frequency of associated problems, validating the notion that intensive use suggests a greater risk of negative consequences. Likewise, the profiles provide a guide for the design and implementation of strategies and interventions to promote digital well-being at the university. Similarly, there is a need to adopt an institutional approach that integrates digital well-being into all its educational programs. The results of this research reveal an emerging public health problem that must be addressed promptly.

### Limitations and future lines of research

One of the limitations of this study is the cross-sectional nature of the research design. This design prevents the establishment of causal relationships between the profiles and the observed consequences. On the other hand, the data are based on self-reports, so they may be subject to social desirability biases or estimation errors. Future studies could consider objective measurements (for example, monitoring applications of the actual use of the device) and adopt a longitudinal design to assess the stability or transition between latent classes over time. Similarly, other relevant variables, such as psychological well-being, self-esteem and academic performance, should be incorporated into the analyses. Finally, the study should be replicated in other universities and sociocultural contexts to validate the generalizability of the identified profiles.

## Conclusions

This study identified and characterized two distinct latent profiles of smartphone users among a sample of university students. The latent profiles were identified as *Moderate and Functional Users* and *Intensive and Problematic Users*. Our findings highlight the heterogeneity of digital behavior and move beyond simple measures of screen time to provide a more in-depth understanding of smartphone use among undergraduate students. The profiles were found to differ significantly across behavioral, sociodemographic, academic, and health-related variables, confirming their practical and theoretical relevance. The identification of these profiles holds significant implications for the design of targeted and effective interventions, suggesting that a one-size-fits-all approach to digital well-being is insufficient. This research contributes to the literature on digital behavior by offering a typology that captures its complexity, which can inform future prevention strategies and educational policies aimed at promoting healthy technology use.
